# Multiparametric ultrasound findings in acute kidney failure due to rare renal cortical necrosis

**DOI:** 10.1038/s41598-021-81690-x

**Published:** 2021-01-21

**Authors:** Paul Spiesecke, Frédéric Münch, Thomas Fischer, Bernd Hamm, Markus H. Lerchbaumer

**Affiliations:** 1 Department of Radiology, Charité - Universitätsmedizin Berlin, Corporate Member of Freie Universität Berlin, Humboldt-Universität Zu Berlin, Campus Charité Mitte, Charitéplatz 1, 10117 Berlin, Germany; 2 Department of Nephrology and Intensive Care Medicine, Charité - Universitätsmedizin Berlin, Corporate Member of Freie Universität Berlin, Humboldt-Universität Zu Berlin, Berlin, Germany

**Keywords:** Urological manifestations, Ultrasonography, Acute kidney injury, Renal replacement therapy

## Abstract

Renal cortical necrosis (RCN) is a rare cause of acute kidney failure and is usually diagnosed on the basis of characteristic enhancement patterns on cross-sectional imaging. Contrast-enhanced ultrasound (CEUS) offers benefits in patients with kidney failure in the clinical setting including the use of a nonnephrotoxic intravascular contrast agent and the fact that it can be performed at the bedside in critical cases. Therefore, the aim of this study is to investigate whether CEUS can reliably identify typical imaging features of RCN. We retrospectively analyzed 12 patients with RCN examined in our department and confirmation of the diagnosis by either histopathology, other contrast-enhanced cross-sectional imaging tests, and/or CEUS follow-up. Assessed parameters in conventional US were reduced echogenicity, loss of corticomedullary differentiation, length and width of kidney, hypoechoic rim, resistance index and in CEUS delayed wash-in of contrast agent (> 20 s), reverse rim sign, maximum nonenhancing rim and additional renal infarction. Furthermore, imaging features in RCN were compared with the findings in renal vein thrombosis (RVT), among them echogenicity, corticomedullar differentiation, hypoechoic rim, RI value, delayed cortical enhancement, total loss of cortical perfusion and enhancement of renal medulla. All 12 patients showed the reverse rim sign, while a hypoechogenic subcapsular rim was only visible in four patients on B-mode ultrasound. A resistance index (RI) was available in 10 cases and was always less than 1. RI was a strong differentiator in separating RVT from RCN (RI > 1 or not measurable due to hypoperfusion as differentiator, *p* = 0.001). CEUS showed total loss of medullary enhancement in all cases of RVT. With its higher temporal resolution, CEUS allows dynamic assessment of renal macro- and microcirculation and identification of the typical imaging findings of RCN with use of a nonnephrotoxic contrast agent.

## Introduction

Renal cortical necrosis (RCN) is a rare cause of acute renal failure and is more prevalent in developing countries with inadequate healthcare systems^1^. In acute RCN, both the glomeruli and tubules become necrotic, while acute tubular necrosis only affects the tubules^2^. Fibrin thrombi in the renal capillaries have been described as a pathologic correlate of RCN^2^. Fogo et al. visualized the coagulative necrosis in histopathologic examples^3^.

While the pathogenesis remains unclear and may be multifactorial, small case series or case reports describe an association with shock, sepsis, and postpartum hemorrhage, which lead to reduced renal arterial blood flow secondary to vascular spasm, microvascular injury, or intravascular coagulation^4–6^. Acute renal failure due to RCN appears to be more common in infants and in perinatal women^1^.

In a case series, Frimat et al. describe the clinical course of 18 patients with RCN due to postpartum hemorrhage^7^. All of the included patients were in need of hemodialysis since acute renal failure developed quickly^7^. Eventually, in all of the patients RCN converged in chronic kidney disease, whereas 6 months postnatal 8/18 patients still were in need of hemodialysis^7^.

Histologically, renal cortical necrosis may affect the whole cortex with or without the medulla or just its subcapsular parts. The necrosis and organ damage is described as irreversible loss of function and poor prognosis^6^.

RCN has typical features that can be demonstrated by cross-sectional imaging such as contrast-enhanced computed tomography (ceCT) and contrast-enhanced magnetic resonance imaging (ceMRI) including a nonenhancing cortical rim that correlates with the histopathological findings^8^. Nonenhancement of the cortical rim with simultaneous enhancement of the renal medulla is characteristic of RCN and is known as the “reverse rim sign”^9,10^. This must not be confused with the “cortical rim sign”, which describes a very thin edge of enhancing, viable renal cortex supplied by collaterals of capsular arteries^9,10^. Nevertheless, biopsy and histopathological examination remain the gold standard in confirming the diagnosis of acute RCN and assessing the extent of vascular damage and identifying the affected renal structures^11^.

Ultrasound (US) with color-coded duplex sonography (CCDS) is the primary imaging technique to assess macrovascularization of renal transplants in the early post-transplant period but has been demonstrated to be especially highly specific for macrovascular problems such as renal artery stenosis or vein thrombosis^12,13^. Contrast-enhanced ultrasound (CEUS) is increasingly being used for evaluation of organ perfusion and lesion characterization, as advocated by the EFSUMB guidelines. Specifically, the guidelines recommend CEUS for differentiating cortical necrosis from renal infarction^14^. Real-time dynamic CEUS depicts microcirculation throughout the kidney and should therefore identify the reverse rim sign to diagnose acute RCN. So far, just a few case reports and the results of a single-center investigation of CEUS in acute RCN in five patients have been published^15^. Thus, the aim of our study is to evaluate CEUS imaging findings in acute RCN in both native kidneys and renal transplants in adult patients.

## Material and methods

### Study cohort

All patients gave written informed consent to anonymized use of their data prior to imaging. This is part of the routine clinical procedure at our department. This retrospective analysis is registered at the local ethical committee of our institution (Ethikkommission der Charité-Universitätsmedizin Berlin, EA1/320/20). All study data were collected in compliance with the principles expressed in the 2002 Declaration of Helsinki. Figures were arranged considering anonymization in order to avoid their affiliation to patient’s identity.

The retrospective analysis included patients who underwent CEUS in our department from 2009 to 2019. Inclusion criteria were: (1) CEUS examination with documentation of sufficient image data and detailed written report of findings, (2) diagnosis of renal cortical necrosis in CEUS, (3) available clinical data, (4) patient age ≥ 18 years, and (5) diagnosis confirmed by biopsy and histopathology or imaging follow-up by either CEUS, ceCT, or ceMRI (in case of cross-sectional imaging one month before or after index examination and in case of CEUS follow-up within 2 months after index examination). Exclusion criteria were: (1) substandard image quality, (2) no clinical data available, (3) no imaging follow-up and missing histopathological confirmation, and (4) proven renal vein thrombosis at the time of index examination.

Using the radiology information system (RIS), we collected the following clinical information: renal or systemic primary disease, oliguria, fever, hypertonia, anemia, secondary hyperparathyroidism, diabetes mellitus, and creatinine value determined closest to the time of the index CEUS examination. Furthermore, the included cases were assessed regarding occurrence of acute RCN in the postpartum period or after renal transplant.

### US and CEUS examination protocol

Gray-scale B-mode imaging of the kidney or kidney transplant was performed using a convex array transducer to assess renal size, echogenicity, and homogeneity.

Standardized CCDS was performed to assess venous outflow and arterial circulation and to calculate resistance indices (RI) for different segmental arteries at the pyeloparenchymal border (generally as a mean value of three measured RI in the upper, middle and lower third of the kidney). Power Doppler imaging was used to identify focal perfusion loss.

CEUS examinations were performed as part of clinical routine using high-end ultrasound systems (Aplio 500/i900, Canon Medical Systems Corporation, Tochigi, Japan; Acuson Sequoia/S3000, Siemens Healthineers, Mountain View, CA, USA; GE Logiq E9, GE Healthcare, Chicago, Illinois, USA) with state-of-the-art CEUS-specific protocols available at the time of examination. All convex transducers employed in study patients were required to be for abdominal use with a frequency range of 1–6 MHz. The very-low-mechanical-index (< 0.1) technique was used to avoid early microbubble destruction. A bolus of 1.2–1.6 mL of ultrasound contrast agent (SonoVue, Bracco Imaging, Milan, Italy) was injected up to three times, if necessary, for example to assess arterial inflow in both kidneys. Following contrast agent injection, the kidney or renal transplant was scanned for at least five minutes to capture the wash-in phase (cine loop of 30 to 45 s) and late contrast phase between two and 5 min after injection of contrast agent. Baseline B-mode US, CCDS, and CEUS were reviewed by a highly experienced radiologist with more than 10 years of experience in CEUS imaging (EFSUMB level 3) in consensus with a second experienced radiologist.

### Cross-sectional imaging

Additional multiphase ceCT examinations (arterial and venous/delayed phase) were performed in a 64–128 detector CT scanner using a standard protocol. The contrast agent was bolus-injected into an antecubital vein at a flow rate of 3.0–4.5 mL/s. Contrast media with an iodine concentration of 350–400 mg/mL were administered and their amount adopted to patient’s body weight, followed by a 50 mL saline flush. The acquisition direction was craniocaudal.

CeMRI was performed at 1.5 T or 3.0 T using phased-array body coils. The imaging protocols comprised T2-weighted (w) standard 2D sequences with and without fat saturation (FS) and T1-w unenhanced 2D sequences with and without FS (including in-phase/opposed-phase technique). T1-w 3D sequences with FS were acquired in breath-hold technique before and during arterial and venous/delayed phases following intravenous administration of Gadolinium-containing contrast agents (body weight adapted; manual or automatic injection at approximately 1–2 mL/s flow rate followed by 40 mL saline flush).

Cross-sectional imaging datasets were used for comparison if the examination was performed within one month before or after CEUS examination with renal cortical necrosis diagnosis. Cross-sectional imaging findings were also reviewed by a highly experienced, board-certified radiologist with more than 20 years of experience in radiological imaging in consensus with a second experienced radiologist.

### Reference standard

Biopsies were taken on clinical indication only. All patients presented with clinically relevant native kidney or allograft post-transplant dysfunction manifesting as an otherwise unexplained increase in serum creatinine (≥ 1.3 mg/dL), proteinuria (≥ 1 g/day), or primary nonfunction in the early phase after transplantation. Pathologic examinations of biopsy samples taken in patients with renal allografts were performed by experienced nephropathologists. The diagnosis of antibody-mediated rejection was based on the presence of circulating donor-specific antibodies and significant allograft pathology according to the definitions of the up-to-date Banff classification^16,17^.

### Differentiation from renal vein thrombosis

The differentiation of RCN from renal vein thrombosis (RVT) was evaluated by comparing the following sonographic criteria between these patients with confirmed RCN and RVT (confirmed by surgery and/or cross sectional imaging and/or angiography): reduced echogenicity, loss of corticomedullary differentiation, hypoechoic rim, RI > 1 or not measurable due to hypoperfusion of interlobar arteries, delayed wash-in of contrast agent (> 20 s), total loss of cortical perfusion in CEUS, and enhancement of renal medulla. The begin of arterial phase in CEUS was described in the EFSUMB guideline for CEUS in non-hepatic applications by Sidhu et al. to start between 10 and 20 s (except lungs and liver) and so a cut-off value of 20 s was used to assess the arterial-wash-in of contrast agent as delayed^14^. The same specific protocols for renal CEUS available at the time of examination as used for RCN were utilized for CEUS in suspicion of RVT.

Patients with RVT of native or transplant kidneys were identified by a systematic search for CEUS reports on examinations performed between 2009 and 2019. For inclusion, confirmation of the diagnosis of RVT by either contrast-enhanced cross-sectional imaging, intraoperative finding, or angiography was necessary.

### Statistical analysis

Continuous variables are reported as median and interquartile range (IQR), and categorical variables are reported as proportion of absolute number (n/N) and percentage.

Categorical variables were compared using the chi^2^ test and two-sided Fisher’s exact test, and both values are given to evaluate possible uncertainties due to the small study cohort. A two-sided significance level of α = 0.05 was defined appropriate to indicate statistical significance. All statistical analyses were performed using the SPSS software (IBM Corp., released 2019. IBM SPSS Statistics for Windows, Version 26.0. Armonk, NY: IBM Corp.).

## Results

### Study cohort

Overall, our systematic query retrieved a total of 18 patients with suspected RCN. After application of the inclusion and exclusion criteria, the retrospective study cohort consisted of 12 patients ≥ 18 years who underwent CEUS of a native kidney (n = 6) or renal transplant (n = 6) between 2009 and 2019. The reasons for exclusion in the six excluded patients were: RCN was diagnosed as differential diagnosis only, the presumed RCN turned out to be caused by a macrovascular pathology (e.g. RVT) and insufficient data storage (e.g. regarding stored cine loops and images)—respectively in two cases each.

Five of the six patients in the native kidney subcohort were postpartum women. The standardized renal CEUS protocol in our institution included B-mode ultrasound, CCDS, and CEUS. Baseline characteristics of the study patients are demonstrated in Table 1.

**Table 1:** Baseline characteristic, comorbidities, and clinical findings in all patients.

Variable	Proportion
Age (years)	52.5 (38.25–60.5)
Female sex	7/12 (58.33%)
Chronic kidney disease	7/12 (58.33%)
Kidney transplant affected	6/12 (50%)
Postpartum period	5/12 (41.67%)
Oliguria	7/12 (58.33%)
Fever	2/12 (16.67%)
Arterial hypertension	9/12 (75%)
Renal anemia	6/12 (50%)
Secondary hyperparathyroidism	7/12 (58.33%)
Diabetes mellitus	3/12 (0.25%)
Major bleeding	6/12 (50%)
Creatinine (mg/dL)	6.83 (5.81–8.65)

Continuous variables are given as median (IQR), categorical variables as absolute/total numbers (n/N) and percentages in brackets.

RCN denotes renal cortical necrosis, IQR interquartile range.

Chronic kidney disease was known in seven patients, six of them in the renal transplant cohort: one chronic glomerulonephritis, one status after rapidly progressive glomerulonephritis (RPGN) with vasculitis, two patients with diabetic nephropathy, one terminal renal failure due to shrunken kidneys (atrophic kidneys of unknown etiology), one congenital vesicoureteral reflux, and one autosomal-dominant polycystic kidney disease (ADPKD).

Median serum creatinine was 6.83 mg/dL (IQR, 5.81–8.65 mg/dL). Overall, four patients underwent renal transplant nephrectomy due to cortical necrosis.

### US and CEUS findings

Imaging findings are summarized in Table 2. B-mode US findings revealed no renal enlargement in any patient with a median kidney length of 10.45 cm (IQR, 9.43–11.80 cm) and width of 4.90 cm (IQR, 4.53–5.98 cm). Resistance indices (available in n = 10 patients, not available in n = 1 patient, not measurable in n = 1 patient) were slightly increased in four patients with a median RI of 0.78 (IQR, 0.60–0.91), while only one case showed an RI of 1 and no patient showed RI > 1. A subtle subcapsular hypoechogenic rim on B-mode US was only visible in four cases despite use of high-end US in all cases.

**Table 2:** US and CEUS findings in 12 patients with RCN.

**US signs**	
Reduced echogenicity	6/12 (50%)
Loss of corticomedullary differentiation	6/12 (50%)
Length of kidney (cm)	10.45 (9.43–11.80)
Width of kidney (cm)	4.90 (4.53–5.98)
Hypoechoic rim	4/12 (33.33%)
Resistance index^a^ (n = 10)	0.78 (0.60–0.91)
**CEUS signs**	
Delayed wash-in of contrast agent (> 20 s)	6/12 (50%)
Reverse rim sign	12/12 (100%)
Maximum nonenhancing rim (mm)	4.5 (3–5.8)
Additional renal infarction	4/12 (33.33%)

Continuous variables are given as median (IQR), categorical variables as absolute/total numbers (n/N) and percentages in brackets.

US denotes ultrasound, CEUS contrast-enhanced ultrasound, RCN renal cortical necrosis.

^a^Eleven patients with available data, while one patient had no measurable RI values.

On CEUS, six patients had a delayed wash-in of the contrast agent (> 20 s). Furthermore, all 12 patients showed a non-enhanced peripheral rim (reverse rim sign) with subcapsular loss of enhancement in the presence of enhancing medullary pyramids. The necrotic rim had a maximum width of 4.5 mm (IQR, 3–5.8 mm). In four patients, CEUS demonstrated renal infarction in addition to RCN. Follow-up CEUS performed in five patients no later than 2 months after the initial examination demonstrated progression of RCN in one case and improved enhancement of the subcapsular rim in two cases.

**Figure 1:**
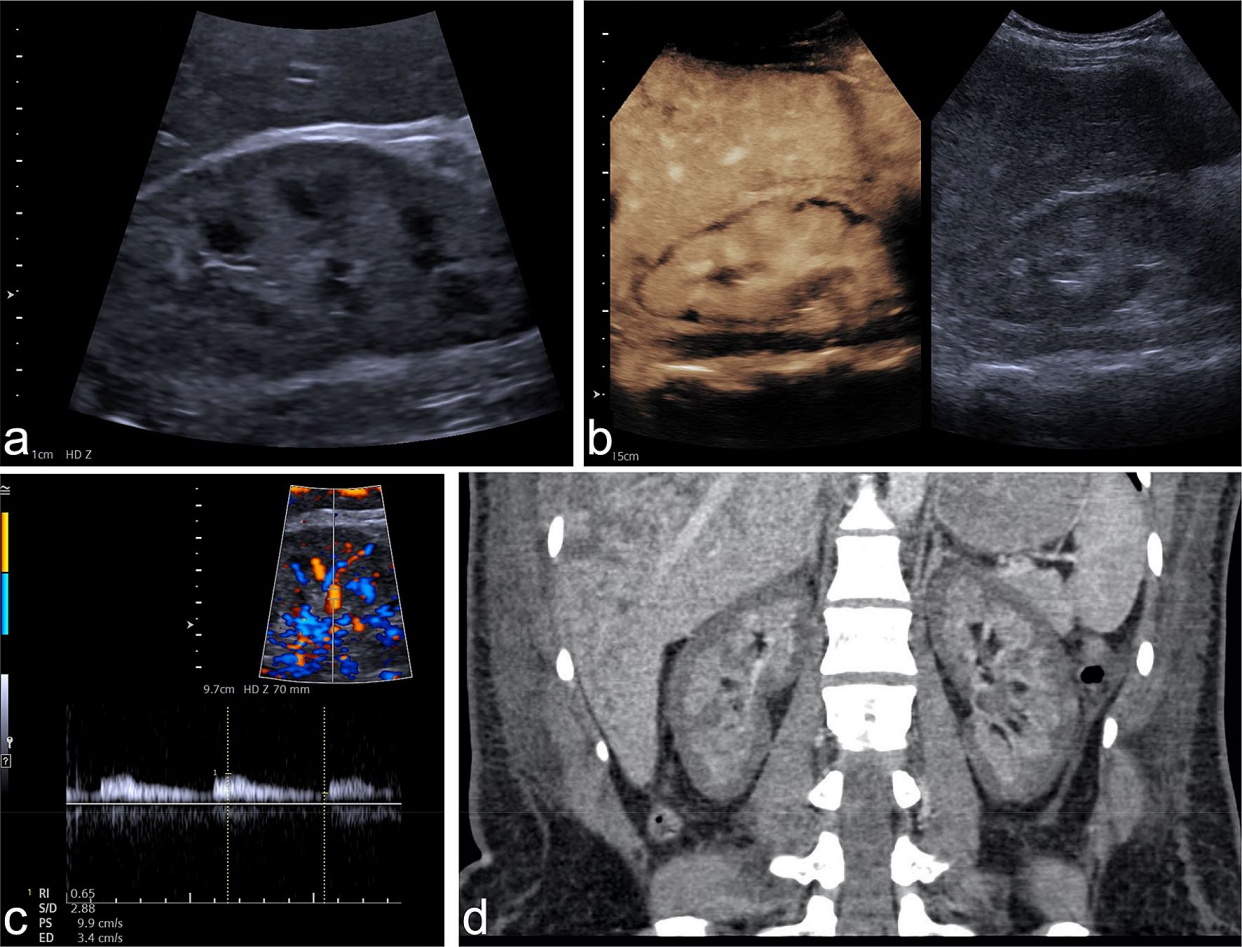
Example of RCN of the native kidney in a 28-year-old woman suffering from massive postpartum bleeding with acute kidney failure and HELLP syndrome. (**a**) B-mode image of the right kidney showing a hypoechoic rim of 3–4 mm. (**b**) CEUS of the right kidney showing a subcapsular loss of contrast enhancement of 3–5 mm. (**c**) Triplex sonography of the right kidney with a PW spectrum of an interlobar artery showing a normal resistance index of 0.65. (**d**) Coronal venous-phase CT scan obtained 14 days before CEUS examination showing a recess of contrast agent measuring up to 6 mm in both kidneys, confirming the diagnosis of RCN. CEUS denotes contrast-enhanced ultrasound, RCN, renal cortical necrosis, HELLP syndrome denotes hemolysis, elevated liver enzymes, low platelets, PW, pulsed-wave Doppler.

**Figure 2:**
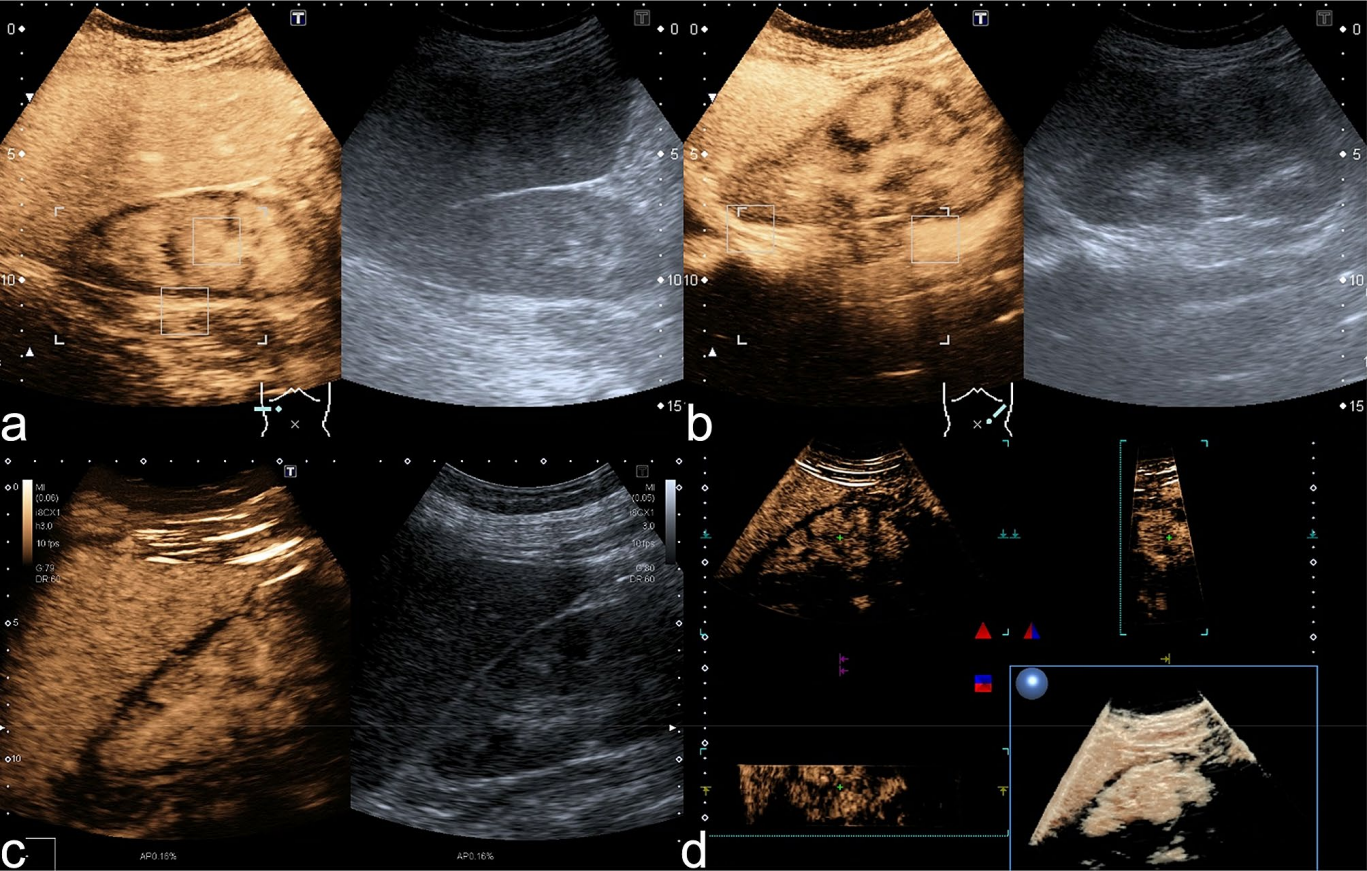
Extent of RCN in CEUS. (**a**) CEUS (0:56 min) of the right kidney in a female patient showing an uninterrupted nonenhancing circular subcapsular rim while B-mode US shows no subcapsular alteration. Note simultaneous enhancement of the medullary pyramids. This pattern is known as the reverse rim sign. (**b**) CEUS (1:20 min) and US image of the left kidney of the same patient consistent with bilateral RCN. (**c**) CEUS (2:15 min) of the right kidney in a different patient showing a subcapsular non-enhancing rim of variable width, especially at the upper pole. (**d**) Same patient as in (**c**). The 3D rendering of the right kidney during CEUS proves the variation in the with of the nonenhancing subcapsular rim.

An exemplary case illustrating the described imaging findings is presented in Fig. 1. Furthermore, Fig. 2 shows examples of different extents of RCN in CEUS.

### Histopathological findings

Overall, a histopathological report was available in seven of the twelve patients (58.3%) included in this retrospective analysis. Six histopathological reports were on renal transplants, and three of them diagnosed cellular rejection. In the other three cases, the diagnoses were: pyelonephritis with ischemic alterations of the cortex, arterial alterations with thrombotic occlusions, and vasculitis. For the only native kidney, for which a histopathological examination was available, the diagnosis was cortical infarction with complete loss of viable cortical tissue. A total of five CD4 immunohistological examinations^17^ were performed and were negative in all cases.

### Cross-sectional imaging

**Figure 3:**
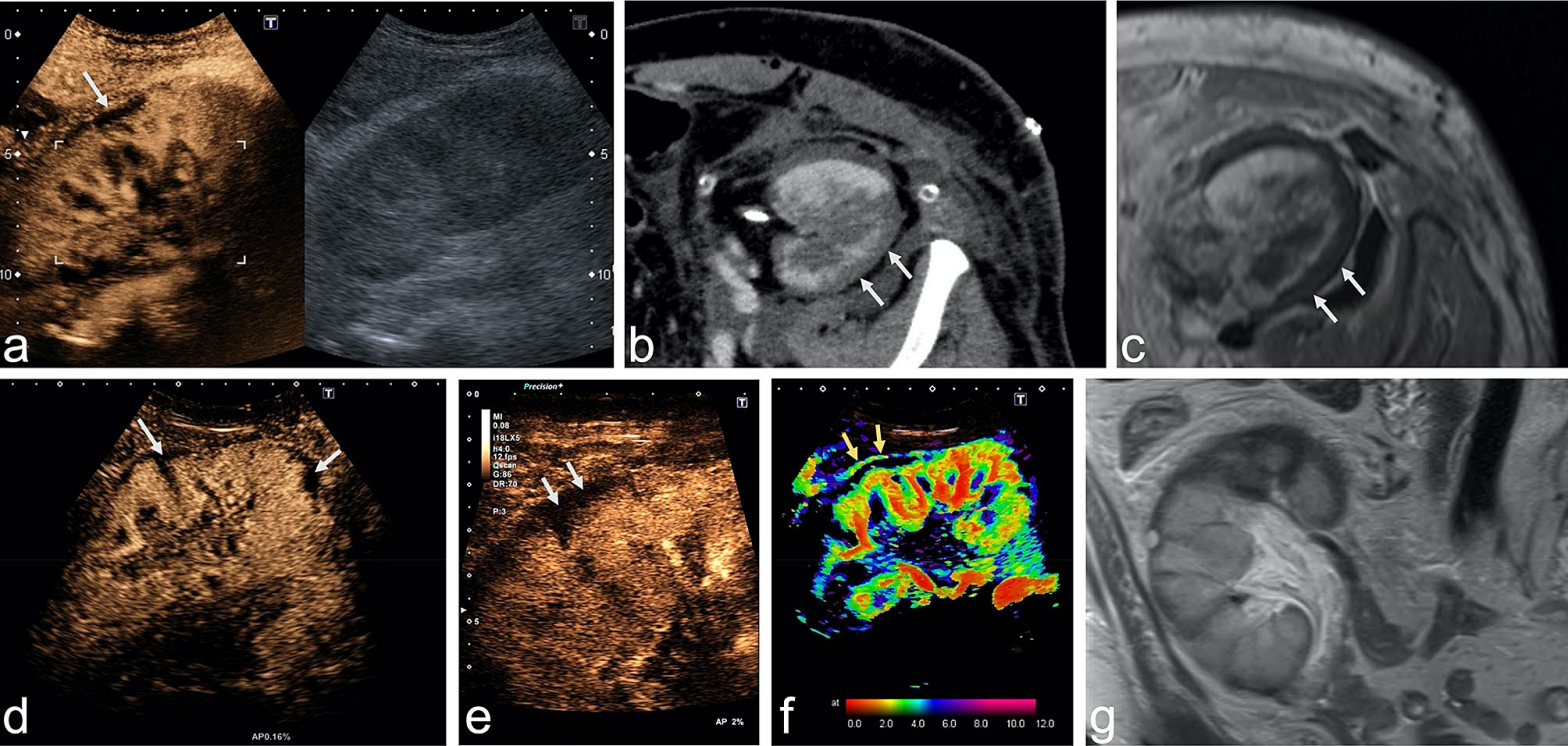
Comparison of modalities in the examination of a kidney transplant with suspected RCN. Upper row: Case showing partial RCN in a renal transplant in a 51-year-old man with end-stage renal failure due to shrunken native kidneys. (**a**) CEUS (0:59 min) and B-mode US of the renal transplant showing typical reverse rim sign with partial loss of enhancement in subcapsular cortex. (**b**,**c**) CeCT (**b**) and ceMRI (**c**) showing the hypoenhanced rim of the renal cortex in the transverse plane. Lower row: A case of RCN in a 54-year-old woman with renal transplant. (**d**) CEUS (1:13 min) using a convex multifrequency probe clearly identifies the reverse rim sign as a nonenhanced rim in the superficial cortex and lower pole (arrows). (**e**) Increased spatial resolution by use of a linear probe, which is important for second look if CEUS with convex probe shows inconclusive findings. (**f**) Parametric arrival time imaging of CEUS depicts arterial inflow in interlobar arteries and cortex within 4 s (red and green). Arrows indicate the so-called cortical rim sign with preserved blood supply by capsular arteries (arrows). (**g**) Coronal T2w MR image confirms the cortical structure defect and moreover shows cortical perfusion deficit at the upper pole of the renal transplant. RCN denotes renal cortical necrosis; CEUS, contrast-enhanced ultrasound; ceCT, contrast-enhanced computed tomography; ceMRI, contrast-enhanced magnetic resonance imaging.

Overall, nine patients underwent additional multiphase ceCT and two patients additional ceMRI, among them one patient who underwent ceCT and ceMRI (Fig. 3). In two patients, RCN detected by CEUS (one native kidney, one renal transplant) was not detected with ceCT. In these two cases, the maximum necrotic rim measured in CEUS was 2 mm and 3 mm. In the two patients who underwent both CEUS and ceMRI, the two modalities showed consistent findings.

Overall, classical reverse rim sign was visible in four of nine patients in case of ceCT and in one of two patients in case of ceMRI. The cortical rim sign occurred only in two patients, respectively one in ceCT and ceMRI.

### Differentiation from renal vein thrombosis

**Figure 4:**
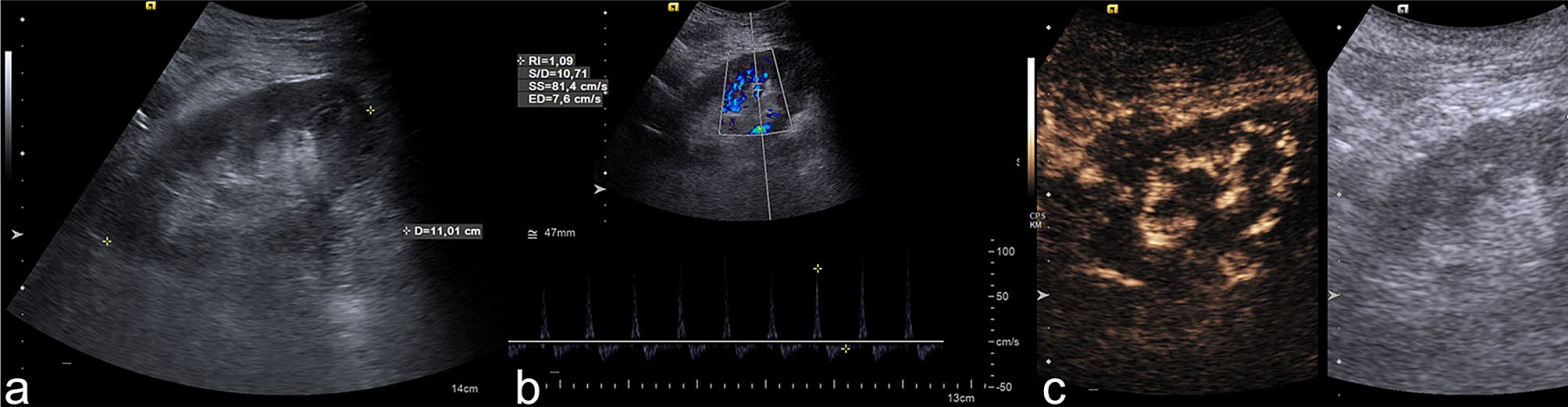
Delimitation of RVT compared to RCN. CEUS examination of a 55-year-old man with renal vein thrombosis. (**a**) The B-mode image shows signs of poor corticomedullary differentiation. (**b**) The pulsed wave (PW) spectrum shows triphasic flow with a resistance index of 1.1, suggesting disturbed venous outflow. (**c**) CEUS (after 50 s) shows no contrast enhancement (indicating loss of microcirculation) of the renal cortex and medullary pyramids. In the cine loop, a pulse-synchronous “pushing” of the microbubbles in the interlobar arteries is visible without cortical enhancement. RVT denotes renal vein thrombosis; CEUS, contrast-enhanced ultrasound, PW, pulsed wave Doppler sonography, RCN, renal cortical necrosis.

Overall, five patients with RVT in CEUS and surgical, angiographic, or cross-sectional imaging confirmation of the diagnosis were included for assessing the ability of CEUS to differentiate RCN from RVT, which is also characterized by a loss of cortical perfusion, however, with preserved thin subcapsular enhancement (rim sign of vascular compromise)^10^. As apparent from the data compiled in Table 3, RI in RVT was always > 1 or not measurable due to significant hypoperfusion of interlobar arteries. Among the eleven RI values available in RCN, only one was not measurable due to hypoperfusion (9.1%) showing therefore strong statistical significance as distinctive feature between RCN and RVT (*p* = 0.001 according Fisher’s exact test). In the differentiation of RCN from RVT, total loss of cortical perfusion was statistically significant in the chi^2^ test but not in the two-sided Fisher’s exact test, whereas enhancing renal medulla (100% in case of RCN, 0% in case of RVT) was found to be statistically significantly different in both tests (*p* < 0.001) compared with RCN. Figure 4 provides examples illustrating the different appearance of RVT in CEUS compared to RCN.

**Table 3:** Comparison of B-mode US and CEUS findings in RCN and RVT.

B-mode US signs	RCN (n = 12)	RVT (n = 5)	*p* (chi^2^)	*p* (two-sided Fisher’s exact test)
Reduced Echogenicity	6/12 (50.0%)	0/5 (0%)	**0.049**	0.102
Loss of corticomedullary differentiation	6/12 (50.0%)	2/5 (40.0%)	0.707	1.000
Hypoechoic rim	4/12 (33.3%)	0/5 (0%)	0.140	0.261
Resistance index > 1 or not measurable due to hypoperfusion	1/11^a^ (9.1%)	5/5 (100%)	** < 0.001**	**0.001**
**CEUS signs**				
Delayed cortical enhancement	6/12 (50%)	4/5 (80.0%)	0.252	0.338
Total loss of cortical perfusion	0/12 (0%)	2/5 (40.0%)	**0.020**	0.074
Enhancement of renal medulla	12/12 (100%)	0/5 (0%)	** < 0.001**	** < 0.001**

A *p*-value < 0.05 indicating statistical significance is marked bold.

Continuous variables are given as median (IQR), categorical variables as absolute/total numbers (n/N) and percentages in brackets.

RCN denotes renal cortical necrosis, RVT renal vein thrombosis, US denotes ultrasound, CEUS denotes contrast-enhanced ultrasound.

^a^Missing image data in one patient.

## Discussion

The main findings of our analysis can be summarized as follows: (1) RI tends to be normal in patients with RCN, (2) real-time CEUS reliably shows the reverse rim sign in RCN, and (3) the combination of CEUS and RI enables differentiation of RCN and RVT in patients with inconclusive CCDS findings.

Imaging findings must always be interpreted in the clinical context and in conjunction with the patient’s history: eleven of the twelve patients with RCN in our study had a renal transplant or were postpartum women. All women with postpartum acute renal failure suffered from massive bleeding, which is characteristic of RCN^1^.

In 2015, Prakash et al. described RCN to be a disappearing entity in developing countries^11^, which makes it even more important to keep this rare clinical picture with its typical imaging signs in mind for correct diagnosis. The importance of the clinical background is further corroborated by the fact that RCN needs to be differentiated from RVT: our results show the RI to be a very strong distinctive feature to separate RCN and RVT. Nevertheless, in inconclusive cases, the use of a contrast agent can be expedient. Our results show—at least using the two-sided Fisher’s exact test—that total loss of cortical perfusion is not clearly statistically significantly different between the two entities, which is probably driven by the small study cohort. Though, the small number of included cases in the comparative group diagnosing a RVT with CEUS is simply explained by the importance of RI in the diagnosis of RVT making the application of contrast agent in most cases needless. Our results indicate this relationship as well since all included patients in the cohort of RVT had a RI value > 1 or the RI was not measurable due to hypoperfusion (Table 3). Next to imaging features, clinical information according the patient are relevant: RVT can occur in acute cases in the post-transplant period and should therefore be considered in these cases^18^. Furthermore, CEUS needs specialized examiners, who are not always available—therefore, a rational use of CEUS in patients with suspected RVT needs to take into account that emergency surgery should not be delayed highlighting the value of CCDS and RI assessment. Nevertheless—showing high importance in macrovascular problems as RVT—a normal RI value should be considered in consensus with contrast-enhanced imaging when suspected RCN, since our results show B-mode sonography assessing reduced echogenicity (50%), loss of corticomedullary differentiation (50%) and hypoechoic rim (33.33%) not to be reproductible in all included patients (Table 2).

External validation of the imaging pattern of RCN in CEUS is very rare. In the largest cohort of five patients with renal transplant reported so far, the investigators described similar results as in our study including an unenhanced subcapsular cortical band and RI not > 1 in all patients^15^. This typical enhancement pattern was also described by Álvarez Rodríguez et al. in two patients and by McKay in a case of bilateral RCN^19,20^. These studies and our results consistently show that the reverse rim sign^10^ is adequately detected by CEUS. The reverse rim sign reflects loss of subcapsular enhancement (Fig. 3a–e, arrows) with preserved medullary enhancement and must be differentiated from the thin cortical rim sign (Fig. 3f, arrows) reflecting preserved supply by capsular arteries. Therefore, CEUS reliably reproduces the classical radiologic signs of RCN which are classically assessed in ceCT^10^. Evidence concerning MRI is much thinner, but was described to be comparable to CT^21^, what could be confirmed in the two patients in our study who underwent ceMRI as visualized in Fig. 3c + g.

Furthermore, there is an ongoing debate about administration of Gadolinium containing contrast agents in patients with kidney disease which is suspected to cause nephrogenic systemic fibrosis (NSF)—which Weinreb et al. summarized to be very low^22^. Therefore, they recommend to balance rarely occurring NSF and potentially delayed diagnosis against each other^22^.

In our analysis, enhancement of the renal medulla was absent in all patients with RVT while it was present in all patients with RCN. Thus, the combination of RI and presence versus absence of medullary enhancement may distinguish between RCN and RVT. Since the absent medullar perfusion as a qualitative parameter showed high statistical significance of *p* < 0.001 (Table 3), it suggests that quantitative evaluation of renal perfusion might not be necessary in clinical practice to differentiate RCN from RVT.

Our study patients had conspicuously high mean serum creatinine levels of 6.83 mg/dL (IQR, 5.81–8.65 mg/dL) as indicators of renal failure. Therefore, CEUS is a suitable imaging method in patients with acute loss of kidney function since, unlike iodine-based contrast agents used in ceCT, ultrasound contrast agent is not nephrotoxic^23–25^. Therefore, CEUS is also suitable for repeat follow-up examinations without a risk of nephrogenic side effects or possible adverse effects of cumulated radiation exposure.

In our study, only 33.33% of the included patients with RCN showed the hypoechoic rim in B-mode ultrasound–although high-end ultrasound machines were used—underlining the need for contrast medium administration in order to diagnose RCN.

In the CEUS follow-up of two patients in our cohort, regression of necrotic areas was observed, indicating that these were cases of incomplete/patchy RCN with the potential of recovery^4,6^. Although partial recovery of renal function has been discussed for such cases before^4^, there was no restoration of renal function in these two patients of our cohort. A similar course was described by Wieler and Hansmann in a case report of bilateral RCN in a 31-year-old patient with recovery of renal perfusion in CEUS follow-up while retention parameters continued to be elevated^26^.

Furthermore, studies investigating focal renal lesions in both native kidneys and renal transplants found CEUS to have higher spatial and temporal resolution than ceCT and ceMRI^27,28^. Since higher resolution also helps in detecting diffuse pathology—especially in renal transplants due to the lower penetration depth in US—CEUS has an important role in these patients. Another important issue are the contrast agents used with different imaging modalities. The microbubbles used for CEUS are strictly intravascular, allowing evaluation whole organ perfusion including microcirculation^29^. This constitutes an advantage for CEUS over both CT and MRI since iodinated and gadolinium-based contrast media are not purely intravascular^29^—which could be especially important when assessing necrotic parenchyma.

## Limitations

We retrospectively analyzed CEUS findings obtained in a small cohort of patients. Since we use a standardized CEUS protocol for clinical examinations in our department, a prospective study design would not have made much of a difference. Furthermore, a retrospective study design is also reasonable given the rareness of the entity investigated. Finally, we used strict inclusion criteria and required a reference standard—either ceCT, ceMRI, CEUS follow-up, or biopsy—for confirmation of the diagnosis.

## Conclusion

Real-time CEUS is a suitable imaging modality for identifying the reverse rim sign in patients with suspected partial RCN, especially when RI values and B-mode US findings are inconclusive. The use of a nonnephrotoxic contrast agent with visualization of renal microvascularization allows evaluation of the entire kidney and use of CEUS for repeat short-term follow-up examinations without a risk of adverse effects on renal function. In combination with RI, CEUS has the potential to differentiate RCN from RVT, which is characterized by complete loss of medullary enhancement.

## Abbreviations

ADPKD: Autosomal-dominant polycystic kidney disease
ANCA: Anti-neutrophil cytoplasmatic antibody
CCDS: Color-coded duplex sonography
ceCT: Contrast-enhanced computed tomography
ceMRI: Contrast-enhanced magnetic resonance imaging
CEUS: Contrast-enhanced ultrasound
NSF: Nephrogenic systemic fibrosis
PW: Pulsed wave
RCN: Renal cortical necrosis
RI: Resistance index
RPGN: Rapid progressive glomerulonephritis
RVT: Renal vein thrombosis
US: Ultrasound
